# The effectiveness of anterior latissimus dorsi and teres major tendon transfers from subscapularis and anterosuperior cuff tears to reverse shoulder arthroplasty: a narrative review

**DOI:** 10.1016/j.xrrt.2025.05.014

**Published:** 2025-06-07

**Authors:** Chang Hee Baek, Jung Gon Kim, Bo Taek Kim, Chaemoon Lim

**Affiliations:** Department of Orthopaedic Surgery, Yeosu Baek Hospital, Yeosu, Republic of Korea

**Keywords:** Irreparable rotator cuff tear, Subscapularis tear, Anterosuperior cuff tear, Loss of internal rotation, Tendon transfer, Latissimus dorsi transfer, Reverse shoulder arthroplasty

## Abstract

Managing of rotator cuff tears, particularly those involving the subscapularis (SSC), anterosuperior cuff, and massive cuff tears with SSC insufficiency, presents significant clinical challenges. Tendon transfers, specifically latissimus dorsi (LD) and combined latissimus dorsi-teres major (LDTM), have emerged as effective surgical options across these scenarios. This review explores the utility and outcomes of these tendon transfers in addressing irreparable SSC tears, anterosuperior cuff tears, as well as massive cuff tears with SSC insufficiency in the context of reverse shoulder arthroplasty (rTSA). This review consolidates existing research on the biomechanics, clinical outcomes, and surgical techniques of LD and LDTM transfers, particularly for SSC tears, anterosuperior cuff tears, and in the context of rTSA for massive cuff tears with SSC insufficiency. The focus was on the effectiveness of these transfers in restoring function and improving clinical outcomes. For irreparable SSC tears, LD transfer showed superior alignment with the transfer principles, effectively replicating the SSC's line of pull, reporting favorable clinical outcomes in short- and long-term follow-ups. In anterosuperior cuff tears, the combined LDTM transfer demonstrated to provide improved joint stabilization by scapulohumeral kinematics with TM and powerful combined 2 tendon transfer, particularly when isolated LD transfer is insufficient. In the context of rTSA for massive cuff tears with SSC insufficiency, the concomitant LDTM transfer demonstrated the capacity to significantly enhance shoulder function, particularly addressing specific deficits in internal rotation compared to rTSA alone. This review underscores the encouraging potential of LD and combined LDTM tendon transfers for addressing complex shoulder pathology, especially irreparable SSC and anterosuperior rotator cuff tears, as well as internal rotation deficits following rTSA in patients with massive cuff tears with SSC insufficiency. While current evidence is promising, further well-designed comparative studies are essential to corroborate these results and optimize their application in complex cases where treatment options are restricted.

The subscapularis (SSC) tendon plays a crucial role in maintaining anteroposterior balance in the transverse plane.^30^^,^^60^ When irreparably damaged, this balance is disrupted, leading to pain and reduced internal rotation strength.^14^ Coupled with supraspinatus insufficiency, it can cause anterosuperior migration of the humeral head due to vertical glenohumeral imbalance, causing anterior shoulder pain and potential progression to pseudoparalysis.^20^^,^^21^^,^^53^ Although less common than posterosuperior tears, anterosuperior tears account for approximately 5%-20% of rotator cuff tear patterns.^35^^,^^60^

Several joint-preserving techniques can address these defects when the articular cartilage is intact.^15^^,^^38^^,^^56^ While superior capsular reconstruction is effective for posterosuperior massive cuff tears, it is not applicable for irreparable SSC tears.^9^, ^10^, ^11^, ^12^, ^13^, ^14^ Anterior capsular reconstruction showed potential but lacks robust clinical data.^51^^,^^54^ Reverse total shoulder arthroplasty (rTSA) restores function but may be less suitable for younger, active patients due to inferior outcomes and high complication rates.^18^^,^^45^^,^^59^

Enhanced understanding of the anatomical and biomechanical properties of periscapular muscles enabled the development of muscle transfer techniques for both posterosuperior and anterosuperior cuff tear deficiencies, including pectoralis major, latissimus dorsi (LD) with or without TM, lower trapezius tendon transfers.^19^^,^^27^^,^^39^^,^^43^^,^^44^^,^^49^^,^^52^^,^^61^ Among these, the LD and latissimus dorsi-teres major (LDTM) transfer has been traditionally used for reconstructing posterosuperior rotator cuff tears for counterbalance by the deltoid and intact SSC, restoring the anterioposterior force couple.^32^ Conversely, the anterior transfer of the LD is increasingly being recognized as a feasible treatment option for irreparable SSC or anterosuperior rotator cuff tears.^39^^,^^43^ Unlike the pectoralis major transfer, which differs significantly in line of pull and does not biomechanically replicate the SSC role, the LD transfer from the posterior chest wall closely replicates it.^24^^,^^39^^,^^61^

In addition, it has been proposed that augmenting the LD tendon transfer with an additional TM tendon transfer, which promotes scapulohumeral kinematics, could enhance joint stability when an isolated LD transfer is insufficient.^24^^,^^26^ This enhanced stability is due to creating a robust posterior line of pull by both muscles. Subsequent biomechanical research and clinical studies on anterosuperior cuff tears have reported positive outcomes with this combined LDTM transfer approach.^4^^,^^9^^,^^10^

Meanwhile, anterior LDTM transfer also showed promise in conjunction with rTSA, particularly for patients with a combined loss of forward elevation and internal rotation.^5^^,^^8^^,^^38^ rTSA reliably improves shoulder range of motion (ROM) and functional outcomes, but its effectiveness in restoring internal and external rotation remains debated.^12^^,^^31^ The anatomical and biomechanical advantages of anterior LD or LDTM transfers suggest that they could be alternatives, especially for addressing internal rotation deficits following rTSA,^5^^,^^8^^,^^38^ in contrast to traditional techniques such as the L'Episcopo procedure, which have been primarily used to manage external rotation deficits.^2^^,^^11^^,^^58^

This review aims to consolidate the latest comprehensive insights on the utility and applicability of anterior LD and LDTM transfers, including a review of the biomechanics and clinical outcomes for anterosuperior cuff tears and the expanded use of these transfers in arthroplasty to improve internal rotation deficits following rTSA.

## LD transfer for subscapularis tendon tear

In irreparable SSC tears, therapeutic interventions have been notably more constrained compared to posterosuperior cuff tears. This limitation is particularly evident in younger, active patients with preserved glenohumeral articular cartilage. The pectoralis major tendon transfer has historically been the most commonly documented surgical procedure for treating these SSC tears.^19^^,^^27^^,^^33^^,^^44^ Initially described in 1997 by Wirth and Rockwood^62^ as a treatment for recurrent anterior glenohumeral subluxation or dislocation in patients with irreparable SSC injuries, this technique has since undergone various modifications. These include using the sternal or clavicular head, or both, of the pectoralis major, along with different techniques for routing of the pectoralis major relative to the conjoint tendon.^36^^,^^41^^,^^48^ While numerous clinical outcome studies have demonstrated improvements in functional scores and patient satisfaction following pectoralis major transfer, several studies have reported variable and unpredictable functional outcomes, and a high failure rate.^16^^,^^25^^,^^28^^,^^29^^,^^37^^,^^62^

**Figure 1:**
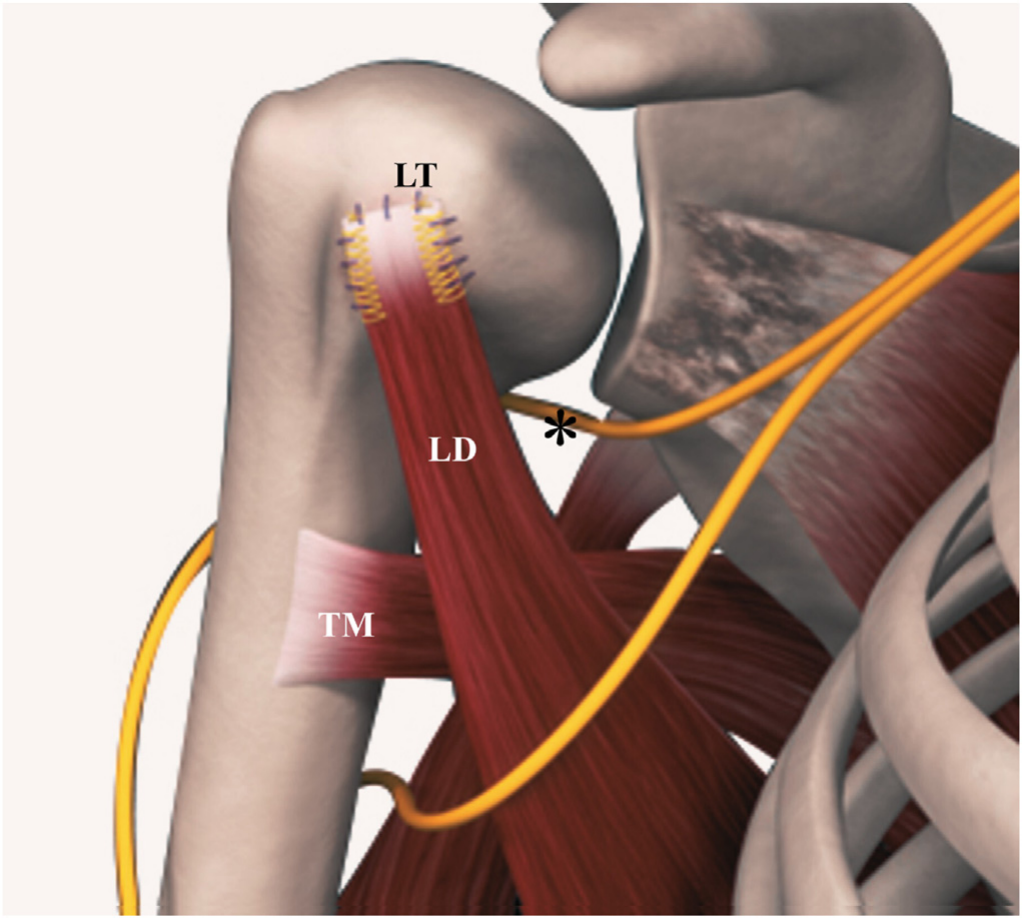
The illustration depicts the transfer and fixation of the latissimus dorsi tendon (LD) to the Upper portion of the lesser tuberosity (LT), while the teres major (TM) tendon remains in its native position, which avoids impingement of the axillary nerve (*∗*).

**Figure 2:**
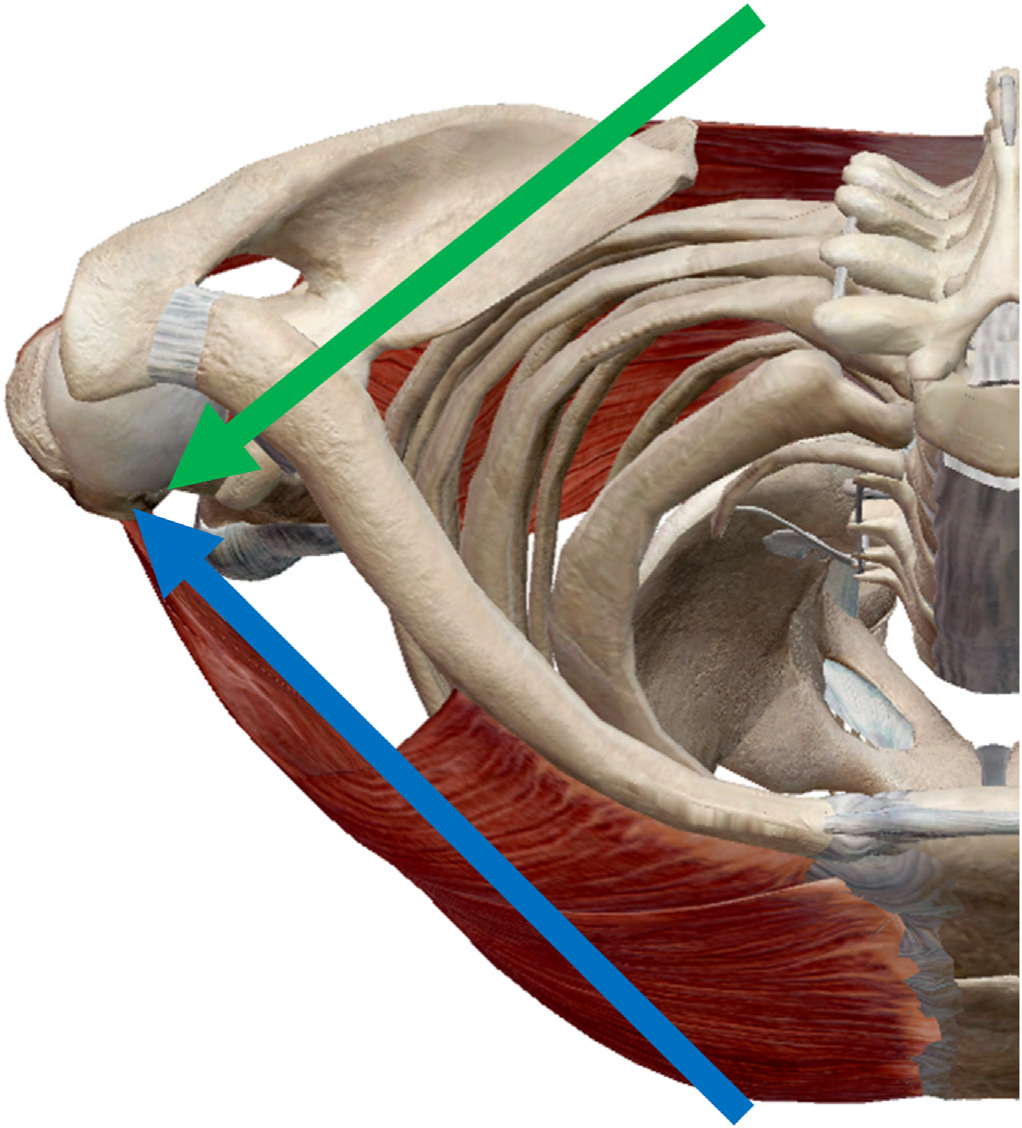
Depiction of internal rotation tendon transfer vectors. The pectoralis major's vector (*blue arrow*) originates from the anterior chest wall, contrasting with the subscapularis arising from the anterior scapular body positioned posteriorly on the chest wall. The vectors for the latissimus dorsi and teres major (*green arrow*), originate from the chest's posterior aspect, in a manner similar to that of the subscapularis. Courtesy of Visible Body.

In recent years, anterior LD transfer has gained recognition as a promising approach for addressing irreparable SSC or anterosuperior rotator cuff tears (Fig. 1).^24^^,^^26^^,^^39^^,^^43^^,^^47^ In contrast, the traditional use of the pectoralis major tendon presents anatomical and biomechanical disadvantages. Originating from the anterior chest wall, the pectoralis major exhibits a line of pull that differs significantly—approximately 90 degrees—from that of the SSC, which arises from the undersurface of the scapula on the posterior chest wall (Fig. 2).^25^^,^^39^ This misalignment violates a fundamental principle of tendon transfer, which emphasizes similarity in the line of pull between donor and recipient muscles.^13^^,^^23^ As a result, the pectoralis major is considered a less ideal candidate for restoring SSC function.^25^

Conversely, the LD demonstrates superior anatomical compatibility. As demonstrated by Elhassan et al,^24^ the LD originates from the posterior scapula, allowing its transfer to better replicate the SSC's native force vector. Moreover, it shares neural origin with the SSC, as both are innervated by branches of the posterior cord of the brachial plexus, further enhancing its synergistic potential.^13^^,^^23^^,^^50^ These anatomical and neurological similarities make the LD a more favorable and biomechanically appropriate option for tendon transfer in irreparable SSC tears.

From a biomechanical standpoint, the LD exhibited superior characteristics to the pectoralis major. A biomechanical cadaveric study was performed to assess and compare the internal rotation moment arm of the pectoralis major, LD, and TM. The research indicated clinical relevance, meaning that the LD transfer might provide a more effective restoration of internal rotation strength and functional outcomes compared to the pectoralis major transfer in clinical settings. Consequently, LD should be considered a viable option for tendon transfer aimed at restoring internal rotation.^61^

In addition, Kontaxis et al^42^ utilized a computational model to analyze the moment arms of tendon transfers of the pectoralis major/minor and the LD, comparing these to an intact SSC. The study demonstrated that the internal rotation moment arms of the transferred pectoralis major and pectoralis minor exhibited a notable reduction after 30° and 40° of internal rotation, respectively, compared to the moment arm of the intact SSC. In contrast, the moment arm of the LD transfer exhibited a closer replication of that of the native SSC throughout the 0°-80° range of internal rotation.

Based on the anatomical and biomechanical strengths of LD transfers, subsequent clinical studies have demonstrated favorable outcomes, though most of these studies are retrospective and lack control groups.^26^^,^^39^^,^^47^ Kany et al^39^ reported on the preliminary outcomes of arthroscopic-assisted anterior LD transfers in a cohort that included 4 patients with failed cuff repairs and one with a failed open Latarjet procedure observed over a minimum of 12 months. They found significant improvements in Subjective Shoulder Value (18.75-63.75) and Constant scores (32.5-68). The belly-press test results improved, becoming negative for 4 out of 5 patients. Only one patient experienced a rupture of the transferred tendon at the final follow-up. In a study conducted by Mun et al,^47^ 24 patients under the age of 65 with massive, irreparable SSC tendon tears were included. A minimum follow-up period of 2 years (with an average of 27.8 months) revealed significant increases in the Mean Constant score (*P* < .001) and the American Shoulder and Elbow Surgeons score (*P* < .001), as well as a reduction in pain (*P* = .006). There were improvements in forward elevation (from 135° to 166°, *P* = .016) and internal rotation (from L5 to L1, *P* = .010), with no significant change in external rotation (*P* = .062). At the final assessment, most patients exhibited negative belly-press and lift-off tests, with no complications or LD retearing reported. Elhassan et al^26^ studied 56 patients undergoing LD tendon transfer, noting significant postoperative improvements in pain, ROM, and shoulder function and clinical scores over an average 13-month follow-up. Notwithstanding these gains, 26% of patients exhibited proximal migration, and 11% developed anterior subluxation, with no significant progression of arthritis. Revision surgeries were infrequent, comprising 2 conversions to reverse shoulder arthroplasty (rTSA) due to tendon ruptures and 3 additional ruptures that did not necessitate further surgical intervention.

More recently, the long-term outcome of LD transfer for SSC tear in 30 patients, with an average follow-up period of 8.7 years, reported for the first time.^6^ The study documented significant improvements in pain, internal rotational ROM, and internal rotational strength. Notably, these benefits were maintained from the short-term to the long-term follow-up. Furthermore, the progression of cuff tear arthropathy remained low-grade. The authors propose that LD transfer represents an efficacious joint-preserving procedure for young and active patients with irreparable SSC tears and present it as a viable alternative to rTSA in nonarthritic patients. In light of these findings, the long-term success of LD transfer is promising particularly when contrasted with the long-term outcomes of pectorals major transfer reported by Ernsbrunner et al,^27^ who, in a longitudinal study spanning an average of 20 years, observed that pectoralis major transfer produces favorable short-term clinical outcomes for both isolated SSC and combined SSC and supraspinatus tears. However, although initial outcomes following surgery indicated a significant improvement in active internal rotation, strength, and SSC-specific examinations compared to preoperative levels, long-term results showed a significant regression to preoperative conditions, with a marked decrease when short- and long-term outcomes were compared.

However, although no comparative studies have been conducted between LD and pectoralis major transfer, and the relative validity of each procedure remains undetermined, the biomechanical advantages of LD transfer—specifically, its line of pull closely aligning with that of the SSC and the reduction of pectoralis major's moment arm during internal rotation—suggest that LD transfer may potentially offer superior clinical outcomes compared to pectoralis major transfer. Nevertheless, to substantiate these distinctions in a clinical context, further longer term well-designed prospective comparative studies should be imperative.

## Combined LDTM transfer for irreparable anterosuperior cuff tear

In situations involving both irreparable tears of the SSC and supraspinatus, often referred to as irreparable anterosuperior cuff tears (IASRCTs), the disruption of the transverse force couple often results in the anterosuperior migration of the humeral head caused by a vertical imbalance within the glenohumeral joint.^14^ In regard to IASRCTs in young and active patients, pectoralis major transfer is a commonly utilized joint-preserving technique. However, it has been documented to result in suboptimal outcomes when compared to isolated SSC tear cases, particularly those that are concomitant with static anterior subluxation and anterosuperior escape.^16^^,^^25^^,^^37^^,^^48^ Elhassan et al^26^ also noted incomplete recovery of superior and anterior humeral head subluxation in cases of IASRCTs after isolated LD transfers; thereby they proposed that incorporating a teres major (TM) tendon transfer, which enhances scapulohumeral kinematics, could improve joint stability when an isolated LD transfer is insufficient.

In a biomechanical analysis, Halder et al^34^ observed that an isolated LD transfer alters the tendon's function depending on the arm's position—specifically, shifting from acting as a humeral head depressor to functioning as a glenoid compressor with increasing forward flexion. This change was accompanied by an increase in scapular tilt at higher abduction angles. In contrast, the TM consistently depresses the head due to its scapular kinematics. A study employing computational modeling by Mulla et al^46^ demonstrated that the TM tendon maintained stable and proportional biomechanical contributions to joint stability and compression throughout humeral elevation, whereas the LD's stabilizing role varied more substantially with positional changes. In addition, the results indicated that the LD and TM tendons effectively served as both joint stabilizers and compressors.

Despite the above biomechanical advantages of combined LDTM transfers, their clinical application must consider potential nerve complications due to the bulky TM muscle. Elhassan et al,^24^ have previously highlighted the close relationship between the TM muscle/tendon and the axillary and radial nerves. Specifically, they have demonstrated that the axillary nerve contacts the TM as it passes through the quadrilateral space. Particularly, increased tension and kinking of the nerve were observed after transferring the TM to the upper part of the SSC insertion. To obviate such complications, they advised against proximal attachment of the TM to the LD. They also recommended that if an isolated LD transfer is insufficient for joint stabilization, an additional separate distal attachment of the TM should be employed, thereby providing enhanced stabilization.

**Figure 3:**
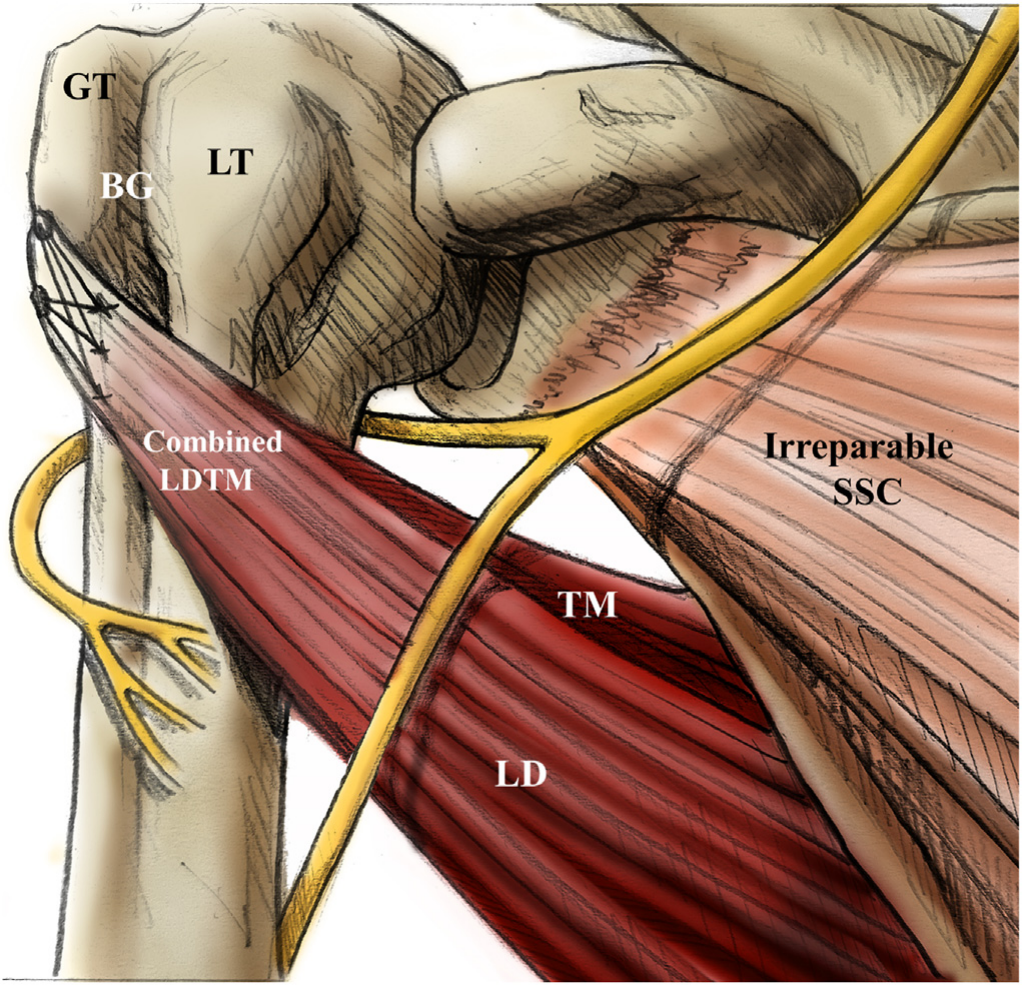
Illustration depicting the secured transfer of the combined latissimus dorsi and teres major (LDTM) tendon to the greater tuberosity (GT). By relocating the attachment site to the GT, the line of pull is altered to a less vertical orientation. Such modification reduces the risk of axillary nerve impingement between the residual inferior portion of the subscapularis (SSC) muscle and the Upper segment of the TM tendon, while also significantly increasing the tension. *LT*, lesser tuberosity; *BG*, biceps groove.

Reprinted from Baek CH, et al.,^4^ with permission from Elsevier.

Baek et al^4^ demonstrated an effective method for circumventing nerve complications by using a modified attachment for combined LDTM transfers. They reattached the LDTM tendon to a modified attachment site that is distally located to the lateral edge of the greater tuberosity and laterally to the biceps groove (Fig. 3). Through this modification, it was explained that not only could axillary nerve impingement by the upper portion of the TM be effectively prevented, but also tendon tensioning could be optimized by attaching it laterally to the biceps groove. This modification was also supported with a biomechanical study by Werthel et al,^61^ demonstrating that transferring the LD or TM tendon to the supraspinatus' footprint enhanced the internal rotation moment arm significantly more than transfers to the SSC's footprint area.

In above clinical study with the modified technique,^4^ they observed notable postoperative enhancements in mean Constant, American Shoulder and Elbow Surgeons, University of California Los Angeles, and activities of daily living requiring active internal rotation scores (all *P* < .001) over a mean follow-up period of 38 months. Furthermore, there was a significant improvement in the active ROM for forward elevation and internal rotation (*P* < .001). In addition, it is noteworthy that only 1 patient experienced transient axillary nerve palsy, which fully resolved with conservative treatment within 3 months.

The author attributed the favorable outcomes and low nerve complications of the combined LDTM transfer to (1) a powerful posterior line of pull enhancing joint stability, (2) using the TM tendon, which consistently depresses the humeral head due to its scapular origin, and (3) reattaching the combined LDTM tendon just distally to greater tuberosity and laterally to biceps groove, which reduces the vertical vector, increases tendon tautness, and avoids axillary nerve impingement. Nevertheless, these clinical findings are exclusively derived from short-term studies conducted at a limited number of institutions. To more accurately ascertain the efficacy of this combined approach, it is imperative to conduct longer-term studies across multiple centers.

Subsequently, a biomechanical study was conducted to evaluate the efficacy of combined LDTM transfer compared to isolated LD transfer in a cadaveric IASRCT model.^9^ The study, which used 8 cadaveric shoulders under IASRCT with isolated LD transfer and combined LDTM transfer attached to the greater tuberosity, confirmed that combined LDTM transfers significantly reduced superior and anteroinferior translations and subacromial contact pressures across all tested conditions. In contrast, isolated LD transfers only significantly reduced superior translation during specific movements and subacromial pressure under limited conditions. Additional biomechanical experiments^10^ using the same conditions and techniques also showed that LDTM transfer resulted in significantly better resting internal rotation—defined as the natural humeral position assumed after release from 60° of external rotation under balanced muscle loading (*P* = .02)—as well as greater maximum internal rotation (*P* < .027), compared to LD transfer in a cadaveric model of anterosuperior rotator cuff tears.

Moreover, recent clinical evidence has supported these theoretical advantages. In a direct comparison between isolated LD and combined LDTM transfers in patients with IASRCTs, Baek et al^7^ reported that the combined transfer group demonstrated significantly superior improvements in activities of daily living that require active internal rotation score, internal rotation strength, and SSC-specific physical examination. These findings suggest that the addition of the TM not only compensates for the potential limitations of the relatively thin LD tendon, especially in smaller or female patients, but also contributes to more balanced scapulohumeral mechanics, thereby enhancing shoulder function in internal rotation.

## Author's preference technique of combined LDTM transfer

Under general anesthesia with an interscalene block, the patient was placed in a beach chair position. Diagnostic arthroscopy assessed the reparability of the SSC and supraspinatus tendons. The LDTM tendon harvest was then initiated. The deltopectoral interval incision was made, extending from the coracoid to the inferior border of the pectoralis major tendon.

**Figure 4:**
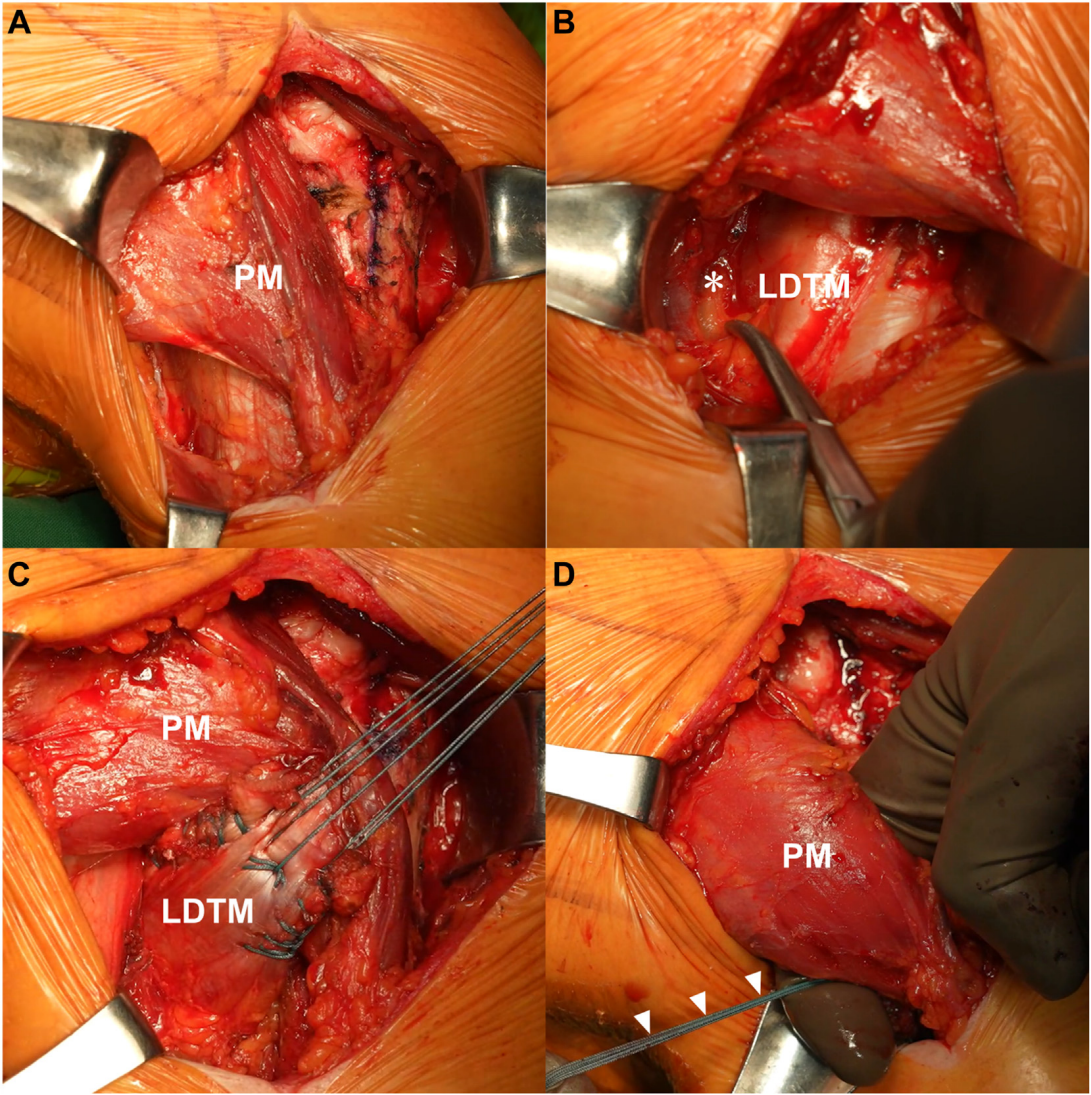
(**A**) Exposure of the dissected and mobilized pectoralis major (PM) muscle, revealing its Upper and Lower borders with unrestricted movement without interference from surrounding tissues. (**B**) The radial nerve running along the posterior aspect of the humerus across the anteroinferior surface of the LDTM, which should be recognized and carefully preserved to avoid iatrogenic injury. (**C**) The prepared harvested combined harvested LDTM tendons with Krakow sutures, which are then completely dissected and released from the adjacent soft tissues. (**D**) The traction suture of the LDTM (*arrow*) is guided beneath the PM muscle using the index finger, as the hook figure method, to thread the harvested LDTM beneath and extract it from under the PM muscle. *LDTM*, latissimus dorsi and teres minor.

In the early stages of LDTM surgery, the procedure involved releasing the proximal third of the pectoralis major tendon at its humeral insertion to expose the tendinous portions of the LDTM tendons adequately. However, current author's techniques favor a pectorals major-sparing approach, where the surface of the pectoralis major is carefully separated from the subcutaneous fat tissue. This separation continues until the pectoralis major muscle's upper and lower borders can be adequately retracted, clearly revealing the underlying LDTM tendon (Fig. 4, *A*). This approach avoids sacrificing the pectorals major, which also serves as an important internal rotator, and is crucial to remain to enhance the internal rotation.

The LD and TM tendons were detached from the humerus in a simultaneous, nonseparated manner. During the harvesting of the LDTM tendon, meticulous care was taken to identify and ensure the protection of the radial nerve, which passes along the posterior aspect of the humerus and beneath the anteroinferior surface of the LDTM tendon. Two long forceps were used to grasp the tendon edges, and nonabsorbable sutures (Ethibond No. 2; Ethicon Inc., Raritan, NJ, USA) were placed in a Krakow fashion. Traction was then applied to the sutures while the surrounding adhesions were bluntly released from the LDTM muscles (Fig. 4, *C*). This procedure enhanced the mobilization and excursion of the muscles.

Subsequently, the nonabsorbable sutures were threaded beneath the pectoralis major muscle using an index finger in a “hook figure” method (Fig. 4, *D*), then drawn toward the greater tuberosity of the humerus. The LDTM muscle was positioned approximately 2 cm distal to the greater tuberosity and lateral to the bicipital groove, aligning the patient's arm in full internal rotation and 45-degree abduction to mirror physiological conditions.

**Figure 5:**
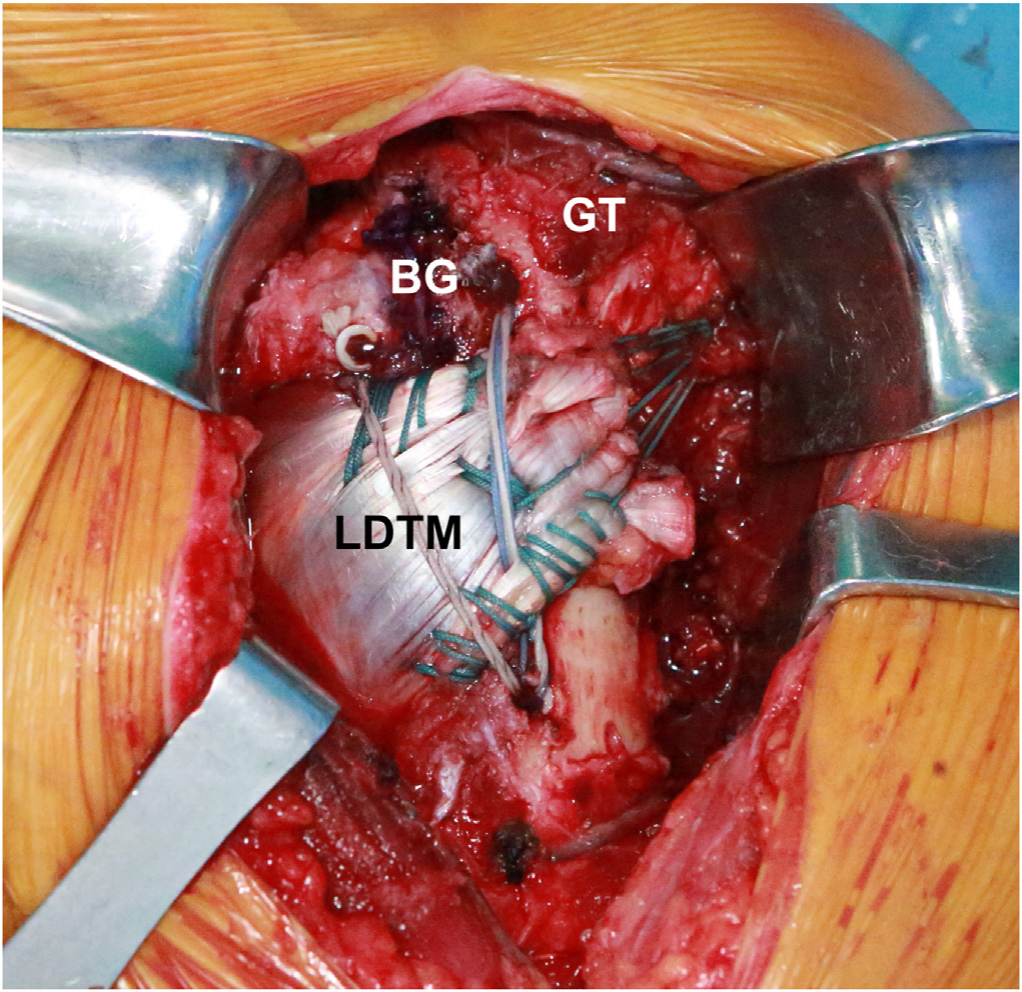
Transfer of the combined latissimus dorsi and teres major (LDTM) tendons attached distally to the lateral edge of the greater tuberosity (GT) and laterally to the biceps groove (BG).

Anchoring commenced with a knotless lateral row anchor (4.75-mm Swivelock; Arthrex Inc., Naples, FL, USA) just lateral to the harvested LDTM to maintain tension. A triple-loaded medial row anchor (4.5-mm Polyether Ether Ketone Corkscrew FT, Arthrex Inc.) was placed beneath the LDTM muscle, lateral to the bicipital groove. Further stabilization involved another lateral row knotless anchor above the LDTM muscle and medial to the bicipital groove, loaded with 4 sutures. An additional lateral knotless anchor secured the remaining 2 sutures above and lateral to the LDTM muscle (Fig. 5).

Alternatively, a transosseous fixation technique can be employed as another method for securing the LDTM tendon. Two parallel bone tunnels are created in the greater tuberosity, spaced approximately 1.5 cm apart, following temporary guidelines drawn from the upper border of the bicipital groove to the superior aspect of the teres minor insertion. A 2.0-mm K-wire is used to drill along these guides. Through each tunnel, #2 nonabsorbable sutures (Permacord; DePuy Mitek, Raynham, MA < USA) are shuttled in a loop fashion with the aid of a spinal needle and loop wire. The leading sutures are then passed retrograde through the tendon to maintain appropriate tension, and each suture is secured with a sliding locking knot positioned at the posterior exit point of the tunnel, providing stable fixation.

The patients were instructed to wear an abduction brace in internal rotation for 6 weeks, with passive ROM and scapular stabilization exercises. Active-assisted ROM began afterward, with restrictions on heavy lifting for 3 months. Full ROM and strengthening exercises commenced at 3 months, and sports activities at 6 months.

## LDTM transfer with rTSA to improve internal rotation strength

rTSA has been demonstrated to be an effective treatment for patients with cuff tear arthropathy and massive rotator cuff tears (mRCTs) who even present with pseudoparalysis.^12^^,^^22^ Despite the ability of rTSA to substantially restore ROM and improve clinical outcomes, the efficacy of the procedure in restoring internal and external rotation remains a topic of debate.^12^^,^^31^ Limited improvement in internal rotation after rTSA has commonly been reported, negatively affecting the execution of daily activities, notably personal hygiene.^55^ This limitation has been considered a crucial factor in determining patient satisfaction and clinical outcomes.^40^

Following rTSA, the joint's center of rotation shifts medially and inferiorly in relation to its position in the native shoulder.^1^ This results in a corresponding shift in the insertion areas of the native internal rotators, including the pectoralis major, LD, and TM, which also move medially and inferiorly. This results in a slackening and reducing these internal rotator musculatures, which inevitably leads to a decreased internal rotation force post-rTSA. Despite advancements in prosthetic design, particularly the introduction of lateralized prostheses, issues of rotational ROM deficits remain unresolved, which continues to present a significant challenge in patients with mRCT and SSC insufficiency with combined loss of elevation and internal rotation.^12^^,^^31^

In this context, although effective treatments for patients with cuff tear arthropathy and mRCT and SSC insufficiency have been lacking, recent biomechanical research has introduced various tendon transfer methods alongside rTSA aimed at enhancing internal rotation.^17^^,^^38^^,^^61^ Werthel et al^61^ analyzed the internal rotation moment arm across different tendon transfers, specifically for LD, TM, and pectoralis major in native shoulder and rTSA settings. The study revealed that LD transfer offers a superior enhancement of IR in native shoulders compared to pectoralis major. Whereas, in cases using an rTSA, the study reported that the biomechanical fulcrum effect provided by the semiconstrained prosthesis allows the pectoralis major transfer to be more efficient in restoring active internal rotation than other transfers. Chan et al^17^ also conducted a biomechanical analysis using cadaveric models to assess the efficacy of rTSA with concurrent tendon transfers for enhancing active rotational ROM. The results demonstrated that the pectoralis major transfer effectively enhanced active internal rotation and performed optimally when combined with a lateralized rTSA prosthesis.

Notwithstanding, previous studies have primarily focused on pectoralis major tendon transfer,^17^ with a paucity of comprehensive evaluations of other tendon transfers for restoring internal rotation. In addition, these studies have typically excluded combined LDTM transfers and concentrated on the internal rotation moment arm rather than directly measuring internal rotational strength.^61^

In contrast to the relatively few biomechanical experiments reported, clinical trials aimed at enhancing internal rotation following rTSA are even more limited. Furthermore, while the usability of rTSA with pectoralis tendon transfer was previously suggested by biomechanical studies, only a limited number of clinical studies have been conducted. These studies primarily focus on surgical techniques and provide only preliminary results^57^ or are limited to single case reports.^3^ Furthermore, as previously stated, pectoralis' major transfer faced challenges as it violated the transfer principle due to the lack of a line of pull comparable to that of the donor in replacement of SSC.^25^

Under these circumstances, biomechanical studies have substantiated the utility of rTSA with LDTM transfer in improving internal rotation. Baek et al,^8^ conducted cadaveric experiments to assess the biomechanical efficacy of anterior LDTM transfer in patients simulating with mRCTs and SSC insufficiency, aiming to enhance postoperative internal rotation. The experiments were performed under 2 conditions: rTSA alone and rTSA combined with LDTM transfer. Internal rotation torque was measured using a torque wrench at 45° and 60° of internal rotation across all abduction angles. In addition, the anterior dislocation forces of the humeral head were measured at 10° of internal rotation and abducted to 20°. Forces were progressively increased until subluxation occurred, and the peak force necessary was documented. Study findings indicated that internal rotation torque was significantly elevated in rTSA with LDTM transfer compared to rTSA alone, achieving 1.43 ± 0.10 Nm vs. 0.80 ± 0.02 Nm across varied abduction and muscle loading conditions. Although rTSA with LDTM transfer required slightly more force to cause anterior dislocation at 91.4 ± 3.9 N against 89.4 ± 4.1 N for rTSA alone, this difference was not statistically significant. These results support the potential reliability of LDTM transfer combined with rTSA as a treatment for patients with mRCT and SSC insufficiency who are expected to experience restricted active internal rotation post-rTSA.

In line with these experimental results, a comparative analysis of the outcomes between patients with mRCT and SSC insufficiency who underwent either lateralized rTSA with or without anterior LDTM tendon transfer has been reported by Baek et al^5^ In this retrospective cohort study, 60 patients with combined loss of forward elevation and internal rotation were enrolled, with 24 patients undergoing rTSA with anterior LDTM transfer and 36 undergoing rTSA alone. With a mean follow-up period of 36.8 months, both groups demonstrated notable improvements in clinical scores and active ROM. However, the LDTM transfer group exhibited superior outcomes in Constant scores, internal rotation activities, and SSC-specific examinations. Nevertheless, ASES scores and forward flexion exhibited comparable outcomes between the 2 groups. The study concluded that, despite the use of lateralized rTSA, patients with cuff tear arthropathy and mRCTs who underwent rTSA with anterior LDTM transfer demonstrated significantly superior clinical and functional outcomes, particularly in activities of daily living that require internal rotation and toileting activities, compared to those who underwent rTSA alone.

Nevertheless, further well-structured clinical studies are needed to compare LD, TM, combined LDTM, and pectoralis major transfers in patients with mRCT and SSC insufficiency across various rTSA prosthesis designs. Furthermore, additional biomechanical investigation is required to ascertain the most efficacious tendon transfer for augmenting internal rotation strength rTSA.

## Author's preference technique of rTSA with combined LD and TM transfer

Similar to the technique described in the prior section, the harvesting of LDTM tendons was performed through a deltopectoral approach using a pectoralis major-sparing method.

The resection of the humeral head was then initiated superior to the junction of the cartilage and the greater tuberosity, following the angles specified by the instrument manufacturer. To optimize press-fit fixation of the humeral stem, the metaphysis and epiphysis were progressively expanded using reamers. Following a complete capsulectomy, the glenoid was prepared. The positioning of the central hole for the glenoid baseplate was facilitated using an anteriorly placed retractor, with the baseplate positioned at the inferior glenoid with a inferior tilt to prevent scapular notching and improve internal rotation.

**Figure 6:**
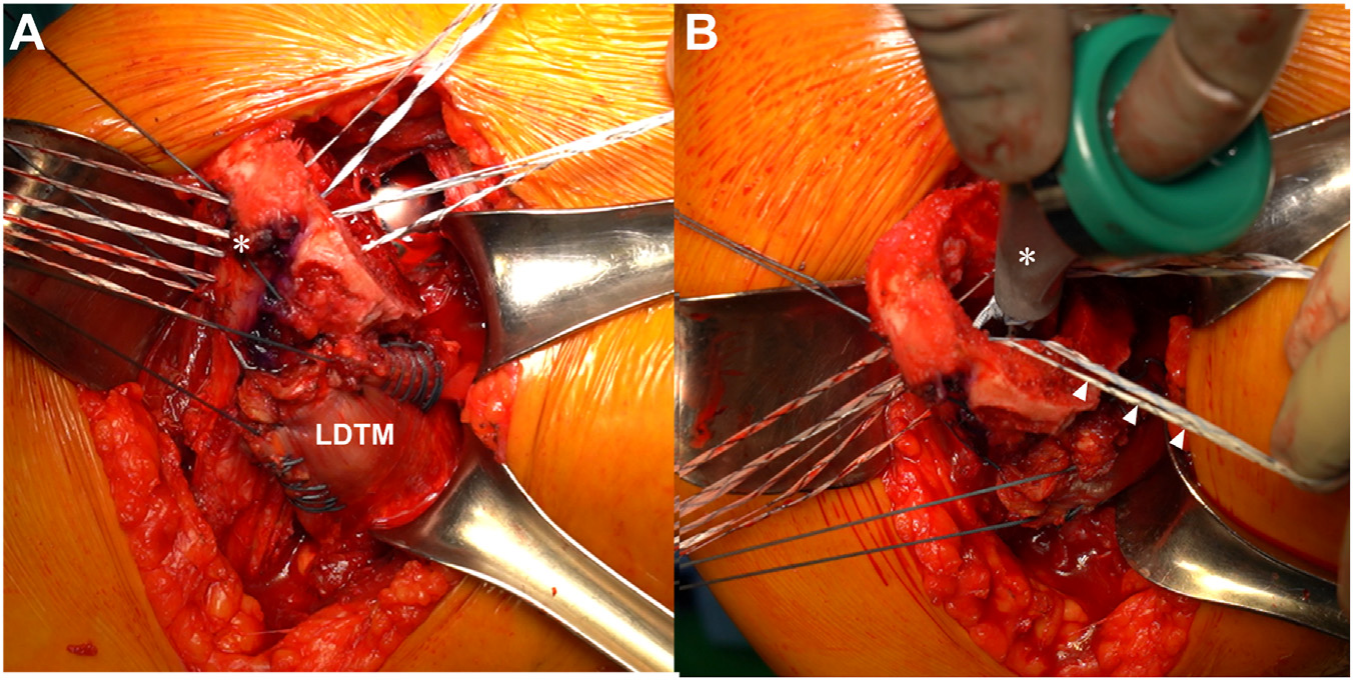
(**A**) Depicting holes created distally to the lateral edge of the greater tuberosity and laterally to the biceps groove and prepared the latissimus dorsi and teres major tendons. (**B**) Showing the stem around looped-suture technique, in which a humeral stem is inserted through a looped suture, thereby providing a secure attachment for the LDTM transfer using these sutures. *LDTM*, latissimus dorsi and teres minor.

**Figure 7:**
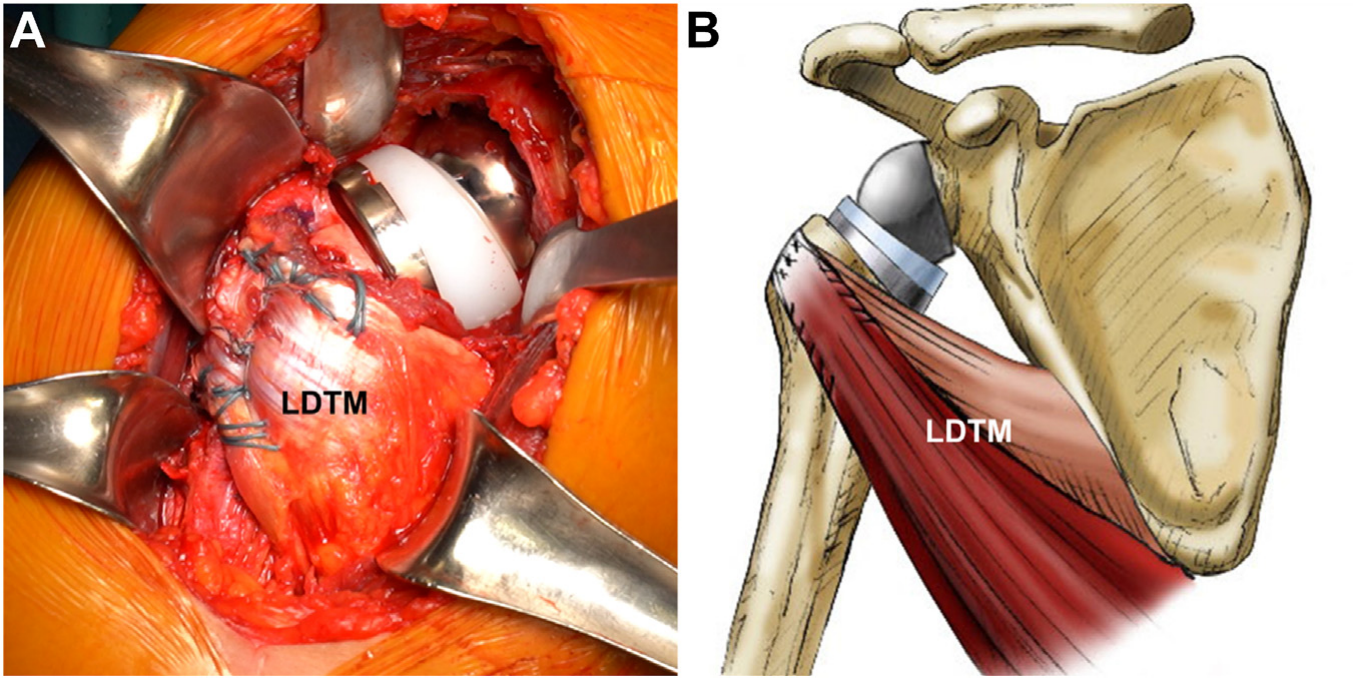
(**A**) Attached tendons at the aspect distally to the lateral edge of the greater tuberosity using the stem-around-looped-suture technique, the transferred tendon is shown firmly secured. (**B**) The illustration depicts the transferred combined latissimus dorsi and teres major tendons, extending from their origin to a modified attachment just distal to the lateral edge of the greater tuberosity following reverse shoulder arthroplasty, thereby avoids impingement of the axillary nerve while effectively tensioning the tendons. *LDTM*, latissimus dorsi and teres minor.

Following the completion of the glenoid procedure, 6-8 holes were created distally to the lateral edge of the greater tuberosity and laterally to the biceps groove. Three to 4 sutures were threaded through the holes mentioned above to form loops. Subsequently, the humeral stem was inserted through the prior-prepared loops (Fig. 6). Before the final impaction, the looped sutures were tied tightly around the humeral stem. The humerus was repositioned onto the glenosphere following the humeral stem insertion. With the patient's arm in full internal rotation and approximately 45° of abduction, the LDTM tendons were tied. The suture looping technique around the humeral stem ensures stable tendon-to-bone healing. In contrast with previous techniques that employed the lesser tuberosity of the SSC footprint, our approach involved fixation of the LDTM tendon transfer in a position distal to the greater tuberosity's lateral edge and lateral to the biceps groove. This resulted in a tendon vector that was less vertical and increased tension, thereby reducing the risk of axillary nerve impingement while optimizing the tendon tensioning effect (Fig. 7).

## Conclusion

This review underscores the noteworthy potential of LD and LDTM tendon transfers in addressing complex shoulder pathologies, particularly irreparable SSC and anterosuperior rotator cuff tears and internal rotation deficits following rTSA in patients with mRCT and SSC insufficiency, capitalizing on their anatomical and biomechanical advantages. While the current evidence is encouraging, further well-structured prospective and multicenter studies are required to fully validate these findings and refine their application in challenging clinical settings where alternative treatments are limited.

## Acknowledgments

The authors extend their gratitude to Sung Hak Choi, Yeong Ran Seo, Seung Hwan Oh, and Seul Gi Yun, who served as research coordinators and provided valuable assistance in collecting clinical and surgical data. The authors acknowledge the use of anatomical images provided by Visible Body for educational and research purposes. Images were accessed and utilized under Visible Body's terms, which permit usage without requiring formal written permission.

## Disclaimers

Funding: No funding was disclosed by the authors.

Conflicts of interest: The authors, their immediate families, and any research foundation with which they are affiliated have not received any financial payments or other benefits from any commercial entity related to the subject of this article.
